# A fast circadian clock at high temperatures is a conserved feature across *Arabidopsis* accessions and likely to be important for vegetative yield

**DOI:** 10.1111/pce.12152

**Published:** 2013-07-09

**Authors:** Jelena Kusakina, Peter D Gould, Anthony Hall

**Affiliations:** 1 Institute of Integrative Biology, University of LiverpoolLiverpool, L69 7ZB, UK; 2 School of Biological Sciences, University of BristolBristol, BS8 1UG, UK

**Keywords:** *Arabidopsis thaliana*, natural variation, performance

## Abstract

The circadian clock is an endogenous 24 h oscillator regulating many critical biological processes in plants. One of the key characteristics of the circadian clock is that it is buffered against temperature, maintaining an approximately 24 h rhythm over a broad physiological temperature range. Here, we tested temperature-buffering capacity of the circadian clock across a number of *Arabidopsis* accessions using several circadian clock reporters: leaf movement, *CCA1 : LUC* and *LHY : LUC*. We found that leaf movement was the best temperature buffered circadian output. On the other hand, when temperature increases, circadian rhythms of *CCA1* and *LHY* transcription shorten considerably across all accessions, indicating that the clock driving expression of *CCA1* and *LHY* is not perfectly buffered. This feature might be crucial to plants growing in a constantly changing environment, and here, we provide insight into the importance of period shortening to plant growth performance and the benefits of a flexible clock.

## Introduction

The Earth’s rotation on its axis results in a 24 h light/dark cycle. The endogenous circadian clock is an adaptation to this cycle, allowing an organism to time events within this 24 h period (Dunlap 1999; Harmer 2009). In eukaryotes and some prokaryotes, a large proportion of critical biological processes are under circadian control, including nitrogen fixation in bacteria, conidiation in fungi, photoperiodic regulation of flowering time in plants and sleep/wake cycles in humans (Pittendrigh *et al*. 1959; Mills, Minors & Waterhouse 1974; Kondo *et al*. 1993; McClung 2001; Albrecht 2002). It is an adaptive advantage to have an endogenous rhythm that matches the periodic environment so that organisms are able to correctly anticipate the time of the day (Ouyang *et al*. 1998; Green *et al*. 2002; Dodd *et al*. 2005). For example, deviation of the photoperiod from the 24 h cycle negatively affects growth of tomato plants, and prolonged exposure of these plants to continuous light significantly damages them (Highkin & Hanson 1954). Furthermore, due to the correct anticipation of dawn, *Arabidopsis thaliana* short- and long-period mutants performed better when their circadian clocks matched the external light/dark environment, fixing more carbon and accumulating more biomass (Dodd *et al*. 2005).

The eukaryotic circadian clock consists of interacting genes and proteins forming interlocking negative/positive feedback loops (Dunlap 1999). According to the current model, the central plant oscillator of the *Arabidopsis* circadian clock is made up of two coupled transcriptional feedback loops, that is, morning and evening (Pokhilko *et al*. 2012). The morning loop involves *PSEUDO RESPONSE REGULATOR 7* and *9* (*PRR7, PRR9*) and *CIRCADIAN CLOCK ASSOCIATED 1* (*CCA1*) and *LATE ELONGATED HYPOCOTYL* (*LHY*) (Alabadi *et al*. 2001; Gardner *et al*. 2006). CCA1 and LHY are MYB transcription factors that regulate *PRR7* and *PRR9*, which in turn inhibit *CCA1* and *LHY*. CCA1 and LHY also bind directly to the *TIMING OF CAB EXPRESSION 1 (TOC1)* promoter from the evening loop and inhibit its transcription, thus coupling the morning and evening loops (Gendron *et al*. 2012; Pokhilko *et al*. 2012).

Temperature massively influences plant growth and survival, and the consequences are especially crucial for commercially grown plants with considerable effects on yield (Law & Crafts-Brandner 1999; Tonsor *et al*. 2008; Zinn, Tunc-Ozdemir & Harper 2010). It is well-known that circadian rhythms are temperature compensated, that is, the clock is well buffered against temperature changes and continues to oscillate with approximately the same period (Pittendrigh 1954). The temperature compensation mechanism is not yet completely understood, but the latest work supports the involvement of *CCA1* and *LHY* in combination with other genes, for example, *GI* (*GIGANTEA*), *PRR7* and *PRR9* (Gould *et al*. 2006; Salome, Weigel & McClung 2010). Due to the high genetic similarity of *CCA1* and *LHY*, it was thought that these clock components have redundant functions in circadian regulation. However, using a *CAB2::LUC* reporter gene in *cca1* null and *lhy* null mutants, it was demonstrated that at different temperatures, the roles of *CCA1* and *LHY* differentiate (Gould *et al*. 2006). Functioning *CCA1* is of more significance in buffering the clock at low temperatures while *LHY* is more important at high temperatures (Gould *et al*. 2006).

Leaf movement assays have previously revealed that there is considerable variation in circadian period and its temperature-buffering capability between *Arabidopsis* accessions (Edwards *et al*. 2005; Michael *et al*. 2003a; Swarup *et al*. 1999; reviewed in Anwer & Davis 2013). Here, we investigated natural variation in circadian clock performance in response to increased temperature by examining two circadian clock genes that have recently been suggested to be members of the clock temperature-buffering mechanism, *CCA1* and *LHY*. We found that the circadian period of *CCA1* and *LHY* transcription shortened at increased temperature. While *CCA1* period shortening was uniform across all accessions, *LHY* period change was accession specific. In addition, preliminary growth performance experiments suggested that a temperature-responsive clock is more beneficial for plants than a temperature-buffered one.

## Materials and methods

### *Arabidopsis* accessions

Seed for all accessions were ordered from the Nottingham Arabidopsis Stock Centre (NASC) or from the Arabidopsis Biological Resource Center (ABRC): An-1 (N6603), C24 (N28126), Col-0 (N1093), Cvi (N8580), Eri (CS22548), Kyo (N3964), Ler (N24596), Sha (N6180), Je54 (N924), Or-0 (N1432), Wc-1 (N1588), Dog-5 (N22699), Phw-1 (N6019), Ct-1 (N6674), Est (N1148), Fei-0 (CS22645), Van-0 (N1585), Ws-2 (N1601). Information on geographical location for all 18 *Arabidopsis* accessions from this study is presented in Table 1.

**Table 1:** Geographical information for the 18 accessions used in the study

Name	Geographical location			
	Country	Latitude (°)	Longitude (°)	Altitude (m)
An-1	Belgium	N 51–52	E 4–5	1–100
C24	Portugal	n/a	n/a	n/a
Col-0	Germany	N 50	E8	1–100
Ct-1	Italy	N 37–38	E 15	1–100
Cvi	Cape Verde Island	N 15–17	W 23–25	1200
Dog-5	Turkey	N 38.3	E 42	1503
Eri	Sweden	N 56.4	E 15.4	n/a
Est	Estonia	N 59	E 26	100–200
Fei-0	Portugal	N 40	W 8	100–300
Je-54	Former Czechoslovakia	N 50	E 15	n/a
Kyo	Japan	N 35.3	E 135.9	n/a
Ler	Germany	N 53	E 15–16	1–100
Or-0	Germany	N 50.5	E 7.6	n/a
Phw-19	UK	N 5l.2	E 0.9	n/a
Sha	Tajikistan	N 39	E 70	3400
Van-0	Canada	N 49–50	W 123	1–100
Wc-l	Germany	N 53	E 10	1–100
Ws-2	Belarus	N 52	E 30	100–200

Information of collection sites was obtained from TAIR (http://www.arabidopsis.org).

n/a, information not available.

The *CAB2::LUC*, *TOC1::LUC*, *CCA1::LUC* and *LHY::LUC* transgenes in pPCV812 were obtained from Lázló Kozma Bognár (McWatters *et al*. 2007). *Agrobacterium tumefaciens* strain GV3101 was used to transform all *Arabidopsis* accessions via the floral dip method (Clough & Bent 1998; Davis *et al*. 2009). Transformed plants were left to recover and senesce. Harvested T_1_ seed was screened on 1.5% agar 1x Murashige and Skoog (MS) plates containing 50 *μ*g mL^−1^ hygromycin. Selected resistant seedlings were allowed to self. Homozygous lines strongly expressing LUC activity were used in experiments with four independent transgenic lines for each construct for each accession.

### Growth conditions

For the leaf movement assays, seeds were surface-sterilized in 70% ethanol for 1 min, 50% bleach with 0.01% Tween 20 for 10 min followed by one rinse in sterile water. For the bioluminescence assays, seeds were gas sterilized for 3 h in a glass desiccation jar holding 500 mL of reverse osmosis water with two dissolved chlorine tablets (CLO-TABS, Arrow Solutions, Swadlincote, UK) and with subsequent addition of 5 mL of hydrochloric acid (modified from Desfeux, Clough & Bent 2000). Seeds were then moved to a sterile flow hood for 1 h to allow any remaining chlorine to evaporate. Sterilized seeds were re-suspended in 0.15% agar and sown onto 1.5% agar MS plates either as individual seeds (for leaf movement) or in rows of eight small clusters of approximately 20–30 seeds (for luminescence). Seeds were stratified in the dark at 4 °C for 3 d before being moved to the 22 °C room and grown under 12:12 light:dark (L/D) cycles of 80–100 *μ*mol m^−2^ s^−1^. Ten-day-old seedlings were used for all experiments.

### Circadian rhythm analysis

For leaf movement, seedlings were imaged in Sanyo MLR350 plant growth chambers (Sanyo, Osaka, Japan) under constant white light (25 *μ*mol m^−2^ s^−1^) at either 17 or 27 °C (depending on the experiment) over the course of 1 week as described by Edwards *et al*. 2005. Images were taken by Sony Exwave HAD cameras (Soverein International, Southport, UK). Luminescence was imaged in a Sanyo MIR-553 incubator (Sanyo Gallenkamp, UK) with an ORCA-II-BT 1024 16-bit camera (Hamamatsu Photonics, Shizouka, Japan) cooled to −80 °C as described in Gould *et al*. 2006. For free-running experiments, LUC activity was monitored at 17 or 27 °C and continuous red/blue light (RBL; 20 *μ*mol m^−2^ s^−1^ of blue light: 20 *μ*mol m^−2^ s^−1^ of red light), provided by red/blue light-emitting diode (LED) arrays (MD Electronics, Coventry, UK). For diurnal experiments, LUC activity of *CCA1*, *LHY*, *TOC1* or *CAB2* reporter genes were monitored at 12, 17 or 27 °C in 12:12 L/D cycles of RBL. Images from leaf movement and luminescence were processed using Metamorph 6.0 image-analysis software (Molecular Devices, Wokingham, UK). Data from the first 24 h after the transfer to continuous light (LL) was not included in analysis. Period estimates and relative amplitude errors (RAE) were calculated in BRASS (available from http://www.amillar.org/downloads.html) by running a fast Fourier transformed non-linear least-square analysis program (Plautz *et al*. 1999). RAE is a measure of rhythm robustness that ranges from 0 (a perfect fit to the wave) to 1 (no fit). Period estimates were used to calculate 1/Q_10_ (Q_10_ is a temperature coefficient of reaction rate change in response to a 10 °C temperature shift) from the equation 1/Q_10_ = (1/*τ*_T+10_)/(1/*τ*_T_), where *τ* and *τ*_T+10_ were period estimates at 17 and 27 °C, respectively. In this study, Q_10_ was an inverse of a real Q_10_ for better visual representation of an increased oscillator speed, which is associated with a period shortening (Edwards *et al*. 2005).

### Plant growth performance assay

Plant performance at 17 and 27 °C was evaluated by determining the dry weight of *Arabidopsis* seedlings. Seeds were sown onto soil, stratified at 4 °C and grown at 22 °C under 12:12 L/D conditions. After 12 d, seedlings were transplanted into 20-cell half trays with one seedling per cell. After 2 d recovery, transplanted seedlings were transferred to 17 °C or 27 °C Sanyo MLR350 growth chambers (Sanyo), with one tray representing each accession at each temperature. After 14 d, prior to bolting, all seedlings were harvested, dried in the 80 °C oven for 48 h and weighed. Weight gain was used to represent the weight increase associated with the 27 °C treatment and was calculated by subtracting the mean weight at 17 °C from the mean weight at 27 °C. Relative weight gain was calculated by dividing the weight gain between 17 and 27 °C by the weight at 17 °C.

### Statistical analysis

Effects of accessions, markers and temperature treatments as well as accession*marker, accession*temperature, marker*temperature and accession*marker*temperature interactions were evaluated using analysis of variance (anova) analysis in MINITAB (Minitab Ltd, Coventry, UK) 15.1.30.0 software. Paired *t*-tests were used to assess the differences between 17 and 27 °C temperature treatments. All calculations were considered to be statistically significant at *P* < 0.05.

### Sequence analysis

Sequences for the nine clock genes *PRR9*, *CCA1*, *ELF4 (EARLY FLOWERING4)*, *GI*, *LHY*, *LUX (LUX ARRHYTHMO)*, *TOC1*, *ELF3 (EARLY FLOWERING3)* and *PRR7* were downloaded for 14 of the phenotyped accessions that had sequenced genomes and had been uploaded to the (http://signal.salk.edu/atg1001/index.php). Sequence analysis was performed using Geneious (http://www.geneious.com). Sequences were aligned using MUSCLE with default settings and for each gene, a phylogenetic tree was drawn using the Jukes Cantor model and the neighbour joining tree-building method with 1000 replicates.

## Results

### Natural variation in the effect of temperature on circadian clock genes

**Figure 1:**
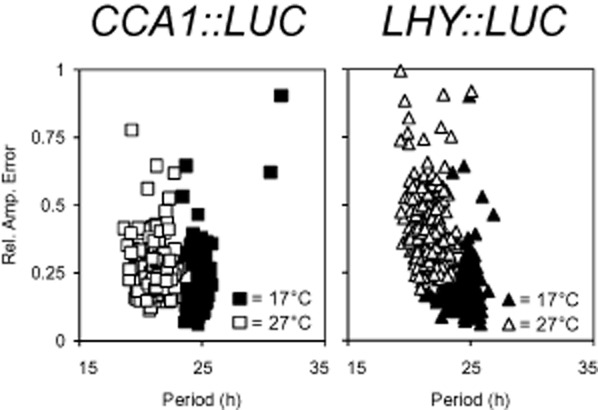
Overview of *CCA1* and *LHY* temperature compensation for 18 *Arabidopsis* accessions. Period estimates for groups of seedlings are plotted against their relative amplitude error (Rel. Amp. Error) with squares representing *CCA1 :: LUC* activity; triangles, *LHY :: LUC* activity; solid symbols, 17 °C data; empty symbols, 27 °C data. Groups of seedlings were grown under 12:12 L/D conditions at 22 °C and after 10 d transferred to continuous light and either 17 or 27 °C where *CCA1 :: LUC* and *LHY :: LUC* luminescence rhythms were assessed. For each accession at each temperature, *n* = 16.

**Figure 2:**
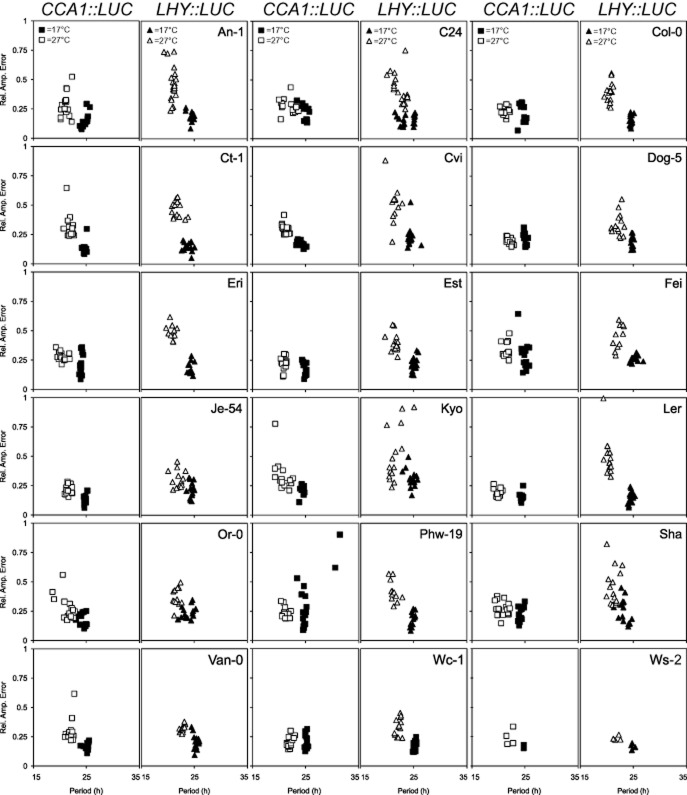
Effect of temperature on *CCA1* (squares) and *LHY* (triangles) in *Arabidopsis* accessions. Plants were grown on MS agar under 12:12 L/D for 10 d before the transfer to 17 (solid symbols) or 27 °C (empty symbols) and continuous light, at which *CCA1 :: LUC* and *LHY :: LUC* rhythms were assessed. Scatter plots illustrate period estimates for each individual group of seedlings plotted against its Rel. Amp. Error. *n* = 16 for all accessions except Ws, where *n* = 4.

CCA1 and LHY are key components of the *Arabidopsis* circadian clock, which have been proposed to have different roles in the temperature compensation mechanism despite their apparent redundancy under standard laboratory conditions (Gould *et al*. 2006). Monitoring expression of these genes should reveal if any variation in temperature buffering of the molecular clock is naturally present in *Arabidopsis* accessions and demonstrate the immediate response of the circadian oscillator to temperature change. *CCA1* and *LHY* transcription was monitored using *LUC* reporter fusions in a variety of *Arabidopsis* accessions (described previously in Millar *et al*. 1995; Table 1). For each accession, four independent transgenic lines of the T_2_ (second generation) were analysed for both *CCA1::LUC* and *LHY::LUC* reporter constructs (normalized average traces, Supporting Information Fig. S1). Prior to the experiment, seedlings were grown at 12:12 L/D and at dawn, they were transferred to continuous light (LL) at either 17 or 27 °C. At 17 °C the circadian period of *CCA1* in tested accessions ranged from 23.9 to 25.2 h with *μ* = 24.7 h (*μ* = period average; Table 2). However, upon transfer of the plants to 27 °C, *CCA1* period became significantly shorter (*μ* = 20.9 h, *t*-test, *P* < 0.001; Fig. 1). No accessions were able to keep the same period of rhythmicity at 27 °C as they did at 17 °C (*P* < 0.001), instead exhibiting 3.1–4.2 h period shortening with the temperature increase, dependent upon accession (Supporting Information Fig. S1). In addition to the period alteration, a slight overall decline in rhythm robustness was observed, with an average RAE value increasing from 0.2 at 17 °C to 0.3 at 27 °C (*P* < 0.001) (Table 2). RAE evaluates rhythm robustness and varies from 0 (robust rhythm) to 1 (no rhythm). Depending on accession, high temperature also induced an increase in the variance of period from 0.64 at 17 °C to 0.86 at 27 °C (*F*-test, *P* = 0.017; Fig. 2). The increase in variance indicates that the precision of the circadian clock is negatively affected by the temperature increase. However, as seen in the RAE plots (Fig. 2), the variance increase was subtle, suggesting that at 27 °C the circadian clock oscillates robustly but with a shorter period.

**Table 2:** *CCA1 :: LUC* and *LHY :: LUC* bioluminescence period estimates (±) and RAE for 17 and 27 °C

Accession	*CCA1::LUC*							*LHY::LUC*						
	17 °C			27 °C			Δ	17 °C			27 °C			Δ
	Period (h)	SE	RAE	Period (h)	SE	RAE	Period	Period (h)	SE	RAE	Period (h)	SE	RAE	Period
An1	25.0	0.1	0.2	20.8	0.3	0.2	4.2	24.3	0.1	0.2	21.1	0.1	0.4	3.2
C24	25.1	0.1	0.2	21.6	0.4	0.3	3.6	23.9	0.5	0.1	21.9	0.5	0.5	2.0
Col-0	24.8	0.1	0.2	20.7	0.1	0.2	4.1	24.5	0.1	0.1	20.5	0.1	0.4	4.1
Ct-1	24.9	0.1	0.1	21.7	0.2	0.3	3.1	23.8	0.4	0.1	21.4	0.1	0.4	2.4
Cvi	24.5	0.1	0.2	20.6	0.1	0.3	3.9	24.3	0.3	0.2	21.7	0.3	0.6	2.5
Dog-5	25.1	0.0	0.2	21.7	0.1	0.2	3.4	24.8	0.1	0.2	21.4	0.3	0.3	3.4
Eri	24.0	0.0	0.1	19.9	0.1	0.3	4.1	24.2	0.1	0.2	20.9	0.3	0.5	3.3
Est	24.9	0.1	0.1	20.7	0.1	0.3	4.2	24.8	0.1	0.1	21.1	0.2	0.4	3.7
Fei-0	25.0	0.2	0.2	21.6	0.1	0.3	3.4	25.2	0.2	0.3	21.9	0.2	0.4	3.3
Je54	24.9	0.0	0.1	21.3	0.1	0.2	3.6	24.6	0.1	0.2	21.8	0.3	0.3	2.7
Kyo	24.6	0.1	0.2	20.5	0.2	0.3	4.1	24.5	0.3	0.3	20.5	0.1	0.4	4.0
Ler	24.3	0.0	0.1	20.0	0.1	0.2	4.3	24.2	0.1	0.1	20.3	0.2	0.5	4.0
Or-0	24.2	0.3	0.2	20.6	0.3	0.3	3.6	23.9	0.2	0.2	21.4	0.2	0.3	2.6
Phw-19	24.7	0.1	0.1	20.8	0.1	0.3	3.9	24.5	0.1	0.1	21.0	0.1	0.4	3.5
Sha	24.0	0.1	0.2	19.9	0.2	0.3	4.1	23.8	0.3	0.2	20.9	0.2	0.4	3.0
Van-0	25.0	0.2	0.1	21.5	0.2	0.3	3.6	25.1	0.1	0.2	22.6	0.1	0.3	2.6
Wc-1	25.2	0.1	0.2	21.8	0.1	0.2	3.4	25.0	0.1	0.2	21.8	0.1	0.3	3.2
Ws-2	24.8	0.0	0.2	21.6	0.1	0.2	3.2	24.7	0.0	0.2	21.4	0.1	0.2	3.3

SE, standard error; Δ, circadian period difference between 17 and 27 °C.

At 17 °C, the *LHY::LUC* circadian period of most accessions was close to 24 h (*μ* = 24.5 h) ranging from 23.8 h to 25.2 h (Table 2). Similar to *CCA1::LUC*, the temperature increase caused considerable period shortening across all accessions (*μ* at 27 °C = 21.3 h; Supporting Information Fig. S1). However, the magnitude of the change was accession specific and ranged from ∼1.9 h (e.g. C24) to ∼4.1 h (e.g. Col-0; Table 2). In contrast to *CCA1*, where seven accessions had more than a 4 h period decrease as the temperature changed from 17 to 27 °C, only three accessions (Col-0, Kyo and Ler) had a ∼4 h period change for *LHY*. This could suggest that the *LHY* rhythm is often better temperature buffered than *CCA1*. On the other hand, temperature had more impact on variance of *LHY* period (period variance increased from 0.75 at 17 °C to 1.03 at 27 °C; *F*-test, *P* = 0.008) than *CCA1* (period variance increased from 0.64 at 17 °C to 0.86 at 27 °C; *F*-test, *P* = 0.017; Table 2; Fig. 2). However, as in *CCA1*, the effect was statistically significant but not very strong (Fig. 2). The 27 °C treatment influenced the robustness of *LHY* rhythms (Fig. 1), where the average *LHY* RAE increased from 0.16 at 17 °C to 0.38 at 27 °C (*t*-test; *P* < 0.001; the average RAE for *CCA1* was 0.15 at 17 °C and 0.25 at 27 °C). Interestingly, in >80% of cases, *CCA1* oscillated faster than *LHY* resulting in a shorter period. Overall, as with *CCA1*, high temperature affected robustness and period of *LHY*; however, the effect on *LHY* was more accession specific. This is consistent with previous reports of functional differentiation of CCA1 and LHY as well as uncoupling of circadian feedback loops when subjected to high or low temperatures (Gould *et al*. 2006).

Analysis of variance was used to determine whether the change in period and rhythm robustness was affected significantly by the circadian marker choice and whether it was accession dependent. It was revealed that RAE was affected by all factors, that is, temperature, accession, choice of marker and their combinations (Table 3). In terms of the circadian period, both markers responded to the increased temperature similarly by shortening the period. However, the effect of the marker*temperature interaction was significant, indicating that there was a marker difference for the temperature treatments used. Indeed, while overall there was no large difference between the molecular markers at 17 °C (*t*-test; *P* = 0.05), the difference became profound at 27 °C (*t*-test; *P* < 0.001), which is well demonstrated in Figs 1 and 2. The accession*marker effect was also significant. For example, in accessions Eri, Ler, Van-0 and Wc-1, *CCA1* and *LHY* oscillated with similar periodicity at 17 °C (*t*-test; *P* = 0.692, *P* = 0.317; *P* = 0.528 and *P* = 0.188, respectively); however, while temperature treatment affected *CCA1* and *LHY* to the same degree in Ler (*t*-test; *P* = 0.084) and Wc-1 (*t*-test; *P* = 0.344), it caused period differentiation between the markers in Eri (*t*-test; *P* = 0.007) and Van-0 (*t*-test; *P* = 0.001). To summarize, even though *CCA1* and *LHY* have a short period at 27 °C in all accessions, the effect that the temperature increase has on each gene is accession dependent.

**Table 3:** ANOVA of *CCA1* and *LHY* circadian period and RAE parameters in *Arabidopsis* accessions

Source	Period (h)					RAE				
	d.f.	SS	MS	*F*	*P*	d.f.	SS	MS	*F*	*P*
Accession	17	193.73	11.40	21.53	0.000	17	1.33	0.08	10.14	0.000
Marker	1	1.07	1.07	2.02	0.156	1	1.45	1.45	188.84	0.000
Temperature	1	2271.02	2271.02	4290.68	0.000	1	4.36	4.36	567.21	0.000
Accession^*^Marker	17	21.46	1.26	2.38	0.001	17	0.41	0.02	3.16	0.000
Accession^*^Temperature	17	85.06	5.00	9.45	0.000	17	0.64	0.00	4.86	0.000
Marker^*^Temperature	1	8.66	8.66	16.35	0.000	1	1.17	1.17	152.35	0.000
Accession^*^Marker^*^Temperature	17	22.75	1.34	2.53	0.001	17	0.51	0.03	3.94	0.000
Error	971	513.94	0.53							
Total	1042									

d.f., degrees of freedom; SS, sum of squares; MS, mean square; *F*, *F*-statistics value; *P*, probability.

### Natural variation in temperature buffering of leaf movement rhythms

**Figure 3:**
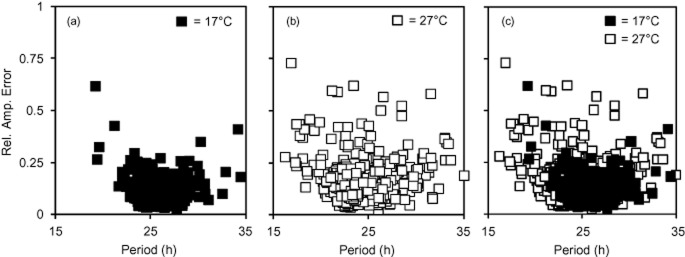
Overview of leaf movement temperature compensation for 18 *Arabidopsis* accessions. Period estimates for individual leaves are plotted against their Rel. Amp. Error with black squares representing 17 °C and white squares 27 °C data. All seedlings were grown on MS agar under 12:12 L/D conditions at 22 °C and after 10 d transferred to continuous light and either 17 or 27 °C where their circadian leaf movement rhythm was assessed. For each accession at each temperature *n* = 30–40. (a) Leaf movement data for 17 °C; (b) leaf movement data for 27 °C and (c) leaf movement data combined for 17 and 27 °C.

We wished to investigate if variation in temperature buffering of the circadian clock observed at the molecular level underlies variation of the overt rhythms previously reported for different *Arabidopsis* accessions (Michael *et al*. 2003a; Edwards *et al*. 2005). We examined temperature buffering of *Arabidopsis* leaf movement rhythms, as it is one of the most common overt rhythms used by circadian researchers. For this, seedlings were grown under 12:12 L/D at 17 °C and after 10 d transferred to LL and appropriate temperature (17 °C or 27 °C), where their free-running circadian periods were measured. On average, at 17 °C all accessions had robust circadian leaf movement rhythms (mean RAE = 0.10) and a range of periods from 24.5 to 27.5 h (Fig. 3; Table 4). As temperature increased to 27 °C, there was a decrease in the circadian rhythmicity robustness (mean RAE = 0.16; Fig. 3). Furthermore, 27 °C caused an increase in the variance of period (from 2.17 at 17 °C to 6.77 at 27 °C, *F*-test, *P* < 0.001) and RAE (from 0.003 at 17 °C to 0.010 at 27 °C, *F*-test, *P* < 0.001), suggesting that the precision of the circadian clock has been affected. In addition to the decrease in the clock precision, temperature increase also caused a general shift towards period shortening (*μ* = 25.7 h at 17 and 23.9 h at 27 °C; *t*-test, *P* < 0.001). This result is consistent with Edwards *et al*. (2005), who also reported an overall period decrease when assaying 27 *Arabidopsis* accessions at 27 °C in comparison to 22 °C.

**Table 4:** Natural variation in leaf movement period (±SE) and at 17 and 27 °C

Accession	17 °C			27 °C			Δ
	Period (h)	SE	RAE	Period (h)	SE	RAE	Period
An1	25.8	0.2	0.1	22.1	0.3	0.2	3.7
C24	24.9	0.1	0.1	24.8	0.5	0.1	0.1
Col-0	25.3	0.1	0.1	24.1	0.4	0.2	1.2
Ct-1	25.2	0.1	0.1	25.2	0.2	0.1	0.0
Cvi	26.0	0.1	0.1	22.9	0.4	0.1	3.1
Dog-5	25.4	0.1	0.1	24.6	0.2	0.1	0.7
Eri	25.0	0.1	0.1	22.9	0.3	0.1	2.2
Est	26.6	0.3	0.1	22.8	0.1	0.1	3.8
Fei-0	27.0	0.1	0.1	23.8	0.2	0.1	3.2
Je54	25.5	0.2	0.1	25.4	0.1	0.1	0.1
Kyo	25.8	0.1	0.1	25.2	0.5	0.2	0.6
Ler	24.9	0.1	0.1	22.8	0.1	0.1	2.1
Or-0	26.2	0.1	0.1	22.8	0.2	0.1	3.4
Phw-19	26.0	0.1	0.1	23.7	0.3	0.1	2.4
Sha	24.5	0.3	0.1	22.9	0.1	0.1	1.7
Van-0	27.5	0.2	0.1	25.8	0.5	0.1	1.7
Wc-1	26.2	0.1	0.1	24.0	0.5	0.2	2.2
Ws-2	26.2	0.1	0.1	24.9	0.4	0.1	1.3

SE, standard error; Δ, circadian period difference between 17 and 27 °C.

**Figure 4:**
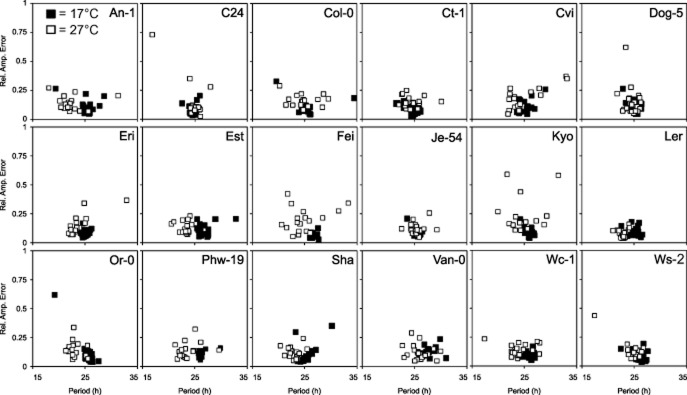
Effect of temperature on circadian leaf movement in *Arabidopsis* accessions. Plants were grown on MS agar under 12:12 L/D for 10 d before the transfer to 17 or 27 °C and continuous light, at which leaf movement rhythms were assessed. Scatter plots illustrate period estimates for each individual leaf plotted against its Rel. Amp. Error. Black squares, 17 °C (*n* = 20); open squares, 27 °C (*n* = 20). Accessions are presented in alphabetical order.

Despite the general period shortening at 27 °C, we found that there was accession-specific variation in this response (Fig. 4; Table 4). Several accessions’ circadian period remained unchanged with the temperature increase (C24, Ct-1, Je54 and Kyo; Table 4). However, unchanged period estimates did not always signify a completely temperature-buffered clock, which is clearly demonstrated by individual RAE plots (Fig. 4). For example, even though the average period for Kyo was statistically the same between 17 and 27 °C, the data on RAE plots appears more scattered. This is illustrated by the spread of data points along the *X*-axis due to the increase in period variance at 27 °C (from 0.52 at 17 °C to 9.16 at 27 °C; *F*-test, *P* < 0.001) in comparison to the tightly clustered points at 17 °C (Fig. 4). Temperature increase caused a significant period shortening in approximately 80% of all accessions tested (14 out of 18) (Table 4). Accessions An1, Cvi, Est, Fei-0 and Or-0 were the most sensitive to temperature, in terms of period shortening, displaying a more than 3 h period decrease when assessed at 27 °C versus 17 °C (Table 4). However, despite the period decrease, temperature did not affect the clock’s precision in An1 (period variance of 3.16 at 17 °C, 7.19 at 27 °C, *F*-test, *P* = 0.09), Est (period variance of 2.98 at 17 °C, 4.11 at 27 °C, *F*-test, *P* = 0.482) and Or-0 (period variance of 2.58 at 17 °C, 1.56 at 27 °C, *F*-test, *P* = 0.312). Conversely, in addition to the period shortening, Cvi and Fei-0 exhibited reduced precision of the clock with period variance changing from 1.71 at 17 °C to 12.09 at 27 °C (*F*-test, *P* < 0.001) for Cvi and from 0.26 at 17 °C to 10.94 at 27 °C for Fei-0 (*F*-test, *P* < 0.001; Fig. 4). In 9 out of 18 accessions, circadian periods changed by 1–2 h and, as previously, the effect on period robustness and clock precision was accession specific (Fig. 4). Overall, natural variation in response to elevated temperature was observed in period and robustness of *Arabidopsis* circadian leaf movement, suggesting accession-specific differences in the circadian clock.

### Increased temperature causes changes in the diurnal expression pattern of key clock genes

Collectively, this study of both rhythms in circadian clock genes and overt circadian rhythms (i.e. leaf movement) has identified a negative relationship between temperature and period length, where period shortens with increasing temperature. A negative relationship between temperature and circadian period has also been observed in cotyledon movement of *Brassica rapa* (Lou *et al*. 2011), suggesting that increases in temperature cause period shortening in plants other than *Arabidopsis*. Period shortening may be an adaptation to warmer temperatures allowing certain clock-controlled aspects of physiology or biochemistry to finish earlier in the photoperiod thus avoiding the hotter parts of the day.

**Figure 5:**
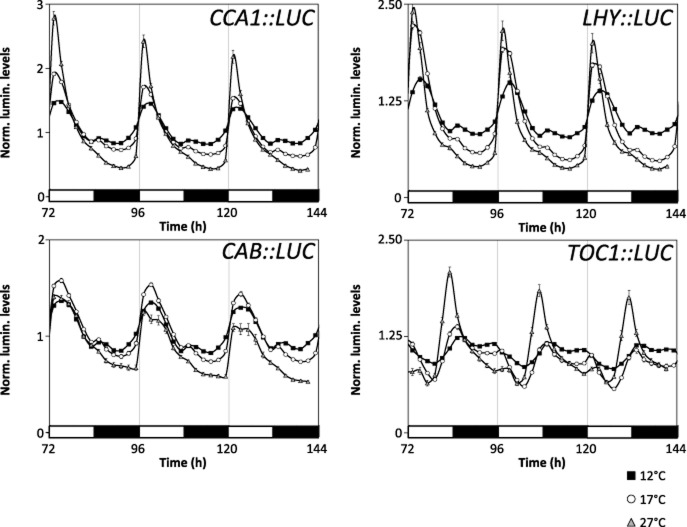
Temperature-dependent changes in the diurnal expression of clock-regulated genes *CCA1*, *LHY*, *TOC1* and *CAB2 .* Transgenic Col-0 seedlings carrying either *CCA1 :: LUC ,* *LHY :: LUC ,* *TOC1 :: LUC* or *CAB2 :: LUC* reporter genes were entrained under 12:12 L/D cycles for 7 d, before transferring to 12, 17 or 27 °C and continued 12:12 L/D cycles. The expression pattern for each marker has been graphed separately with plotted lines representing expression at 12 (black squares), 17 (empty circles) and 27 °C (grey triangles). The plots represent an average of at least three independently transformed lines. The experiment was repeated three times with the data shown here being a representative of the results gained.

While analysis of free-running rhythms allows us to identify small changes in period in the absence of other factors perturbing the rhythms, another important question is how do changes in free-running period alter the diurnal expression of clock-regulated genes? To investigate this, plants of the Col-0 background expressing *LHY*, *CCA1*, *TOC1* or *CAB2::LUC* were grown under 12:12 L/D at 22 °C. They were then transferred to 12, 17 or 27 °C, with the 12:12 L/D continued. Both of the morning genes, *LHY* and *CCA1*, displayed a rapid and sharper increase in their promoter activity following dawn at 27 °C compared to 12 and 17 °C (Fig. 5 and Supporting Information Fig. S2). In addition, the trough in their expression was lower. For *LHY::LUC*, there is also a much lower level in expression at 27 °C with its peak being of a similar level to trough expression at 17 °C (Supporting Information Fig. S2). *CCA1::LUC*, unlike *LHY::LUC*, shows very similar peak expression across temperatures. *CAB2*::*LUC,* like that of *CCA1* and *LHY*, also shows a rapid and sharper increase in promoter activity at dawn for 27 °C data compared to 12 and 17 °C. *CAB::LUC* at 27 °C can be seen to have much higher promoter activity but due to dampening becomes lower than that of 17 °C by the end of the time-lapse experiment. For *TOC1*,a high peak of promoter activity was observed at dusk at 27 °C, which is consistent with the decreased levels of *TOC1* repressors, LHY and CCA1. To further analyse this, we calculated the maximal phase of expression at 17 and 27 °C relative to that of 12 °C for each marker separately. Phase changes occurred for both *TOC1* and *LHY* at 27 °C only, showing a 2 h phase advance (Supporting Information Fig. S3). No phase changes occurred across the temperature range of 12–27 °C for either *CAB2* or *CCA1*. In addition to phase changes, a striking change in shape can be seen for *TOC1* (Fig. 5) with a second peak occurring at the dark to light transition, which is enhanced as temperature increases. Interestingly, this second peak showed a ∼6 h phase delay at 27 °C and a ∼2 h phase delay at 17 °C when compared to 12 °C.

Together, these results are in agreement with a temperature-dependent change in the expression pattern of clock genes under diurnal conditions. They would also support the idea that a faster clock at higher temperatures may shift aspects of physiology and biochemistry earlier in the day.

### Variation in growth performance in high temperature

Based on work from Dodd *et al*. (2005), which demonstrated that plants with an endogenous circadian period closely matching the natural day length outperform plants with a period mismatching against the external day length, we can predict that plants with well temperature-compensated clocks would perform best at higher temperatures. However, our observation, that the molecular clock period shortens at high temperature, is a feature conserved in all tested accessions of *Arabidopsis* and in *B. rapa* (Lou *et al*. 2011), and may point to the importance of a flexible clock capable of subtle adjustments in period. These changes could in turn coordinately regulate whole sets of genes subsequently affecting plant performance.

All *Arabidopsis* accessions examined here possessed clocks with a range of different temperature-buffering capacities. To check whether temperature-related changes in clock period could be of immediate importance to plant performance, we analysed the growth performance of each accession. For this performance study, we chose to measure total aerial biomass, as it is a strong overt indicator of plant growth performance (Dodd *et al*. 2005). Seedlings were germinated in compost and grown under 12:12 L/D at 22 °C for 12 d before being transplanted into individual 20-cell trays. After an additional 2 d at 22 °C, the trays were moved to 17 or 27 °C. On the 14th day, just before plant bolting, all plants were harvested and dried. Dry biomass for each seedling from every accession was later determined.

**Figure 6:**
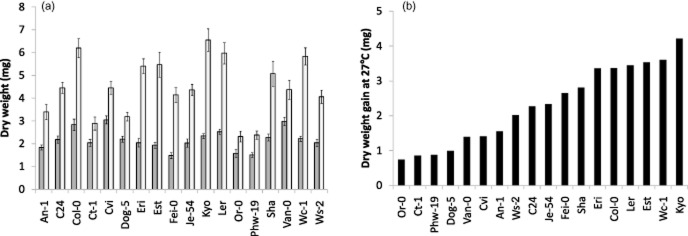
Natural variation in *Arabidopsis* growth performance at 27 °C. (a) Dry weight measured at 17 (dark bar) and 27 °C (light bar); (b) evaluation of dry weight gain at 27 °C. Seedlings were grown at 22 °C and 12:12 L/D for 12 d before transplanting to individual cell trays. After 2 additional days at 22 °C, all trays were moved to 17 or 27 °C. All plants were harvested after 14 d. Weight gain was calculated by subtracting the 17 °C weight mean from the 27 °C mean. For b, accessions are arranged from the smallest to the largest value.

We found that high temperature promoted growth in all accessions, but there was considerable variation in growth response between them (Fig. 6). At 17 °C, size and weight varied considerably between accessions. Therefore, weight gain between the 17 and 27 °C, rather than plant absolute weight, was used to represent performance data. The dry weight gain between 17 and 27 °C was accession dependent and varied from ∼0.75 mg to >4 mg. Accessions Or-0, Ct-1, Phw-19 and Dog-5 had a small weight gain of less than 1 mg (Fig. 6), suggesting that their growth is less influenced by high temperature. In comparison, Eri, Col-0, Ler, Est, Wc-1 and Kyo were the accessions most responsive to 27 °C temperature, exhibiting more than 3 mg dry weight increase.

**Figure 7:**
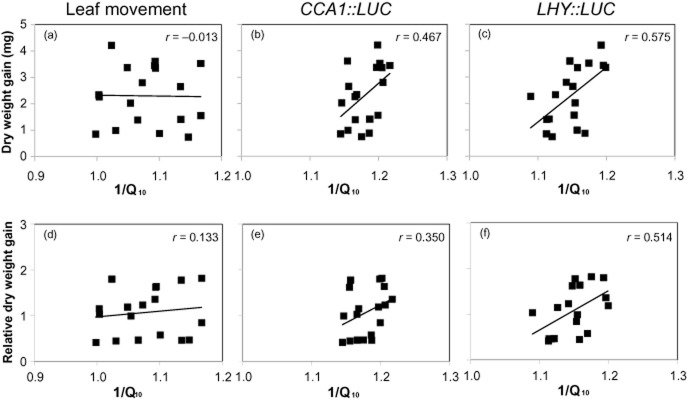
Relationship between growth performance and temperature compensation of leaf movement (a, d) *CCA1* (b, e) and *LHY* (c, f). Seedlings were grown at 22 °C and 12:12 L/D for 12 d before transplanting to individual cell trays. After 2 additional days at 22 °C, all trays were moved to 17 or 27 °C. All plants were harvested after 14 d, dried in the 80 °C oven for 48 h and weighed. 1/Q_10_ was calculated from the equation 1/Q_10_ = (1/T_27_)/1/T_17_), where T_17_ and T_27_ were period estimates at 17 and 27 °C, respectively. 1/Q_10_ is used for better visual representation of an increased oscillator speed, which is associated with a period shortening. *r*, correlation coefficient. (a, b and c) Weight gain was used to represent the weight increase associated with the 27 °C treatment and was calculated by subtracting the mean weight at 17 °C from the mean weight at 27 °C. (d, e and f) Relative weight gain was calculated by dividing weight gain between 17 and 27 °C by weight at 17 °C.

To investigate the relationship between plant performance and temperature-buffering capacity of the clock, we analysed plant dry weight gain and Q_10_ for each of our circadian markers (leaf movement, *CCA1* and *LHY*) for each accession. We found no correlation between the temperature-buffering capacity of leaf movement period and dry weight (Fig. 7a,d). Furthermore, no correlation existed between weight change and rhythm robustness of leaf movement (data not shown). This result was surprising, as leaf movement is the most buffered against temperature changes (Fig. 3). Interestingly, a clear trend was revealed between *LHY* temperature compensation and both raw growth performance data and data normalized to the 17 °C weight with correlation coefficients (*r*) of 0.575 (*P* = 0.013) and 0.514 (*P* = 0.029), respectively (Fig. 7c,f). The interaction between weight and *CCA1* temperature compensation was also significant when using the raw performance data (*r* = 0.467; *P* = 0.051), although this becomes slightly non-significant when normalized to 17 °C weight (*r* = 0.350; *P* = 0.154; Fig. 7b,e). These data imply that accessions with circadian clocks that are poorly buffered against temperature, that is, having greater period difference between 17 and 27 °C, gain more weight at high temperature, while growth of those with well-buffered clocks is compromised. Therefore, it appears that a flexible endogenous clock might be more advantageous than a perfectly buffered clock.

### Sequence variation of core clock genes across accessions

It is entirely possible that evolution has selected specific components within the clock to achieve optimum temperature-dependent period changes that are best suited to the environment. In *Neurospora crassa*, natural variation in circadian period and temperature compensation has been linked to the length of the activation domain of the WC-1 protein, more specifically, to the number of simple sequence repeats (SSRs) present in the NpolyQ (amino-terminal polyglutamine domain) region (Michael *et al*. 2007). It is possible that a similar molecular source causes phenotypic circadian variation in temperature compensation in *Arabidopsis* accessions.

To try and identify such molecular components underlying phenotypic circadian variation in temperature compensation, we have utilized data generated from the 1001 genome project. We downloaded sequence data for all the key clock genes *PRR9*, *CCA1*, *ELF4*, *GI*, *LHY*, *LUX*, *TOC1*, *ELF3* and *PRR7*, for 14 of our phenotyped accessions. The sequences included the complete coding region, together with 1 kb of upstream sequence. The genes were aligned and used to make phylogenetic trees (Supporting Information Fig. S4). From this analysis, clear clusters of accessions could be seen for different clock genes. This was most pronounced for *PRR7* where the accessions could be divided up into two clear haplotypes. These two haplotypes have been previously suggested as responsible for a period QTL between Ler and Col-0 (Michael *et al*. 2003a). However, we failed to find a correlation between clustered genotypes with clusters of similar temperature-dependent clock phenotypes.

## Discussion

In this study, we show natural variation in temperature buffering of the circadian clock and a clear trend in period shortening of the clock in response to increasing temperature. We demonstrate that temperature-dependent period shortening in free-running conditions may have consequences for the temperature-dependent changes in expression patterns we observe for clock genes in diurnal cycles. Finally, we demonstrate a correlation between decreased temperature-buffering capacity and enhanced performance, suggesting that a shorter period at higher temperature may confer a performance advantage.

Natural variation in temperature compensation of leaf movement rhythms has previously been described (Edwards *et al*. 2005). Here, we identified variation in the temperature response of two key clock genes, *CCA1* and *LHY*. While temperature caused a more uniform effect on the circadian period of *CCA1* across accessions, temperature effects on *LHY* period were more accession specific. Therefore, our study has uncovered temperature-dependent differences in the coupling of these two key clock genes, suggesting that at higher temperatures in some accessions, these two genes may be driven by different oscillators. Similar period differences in a single plant have been reported for *CAB2* and *PHYB* (phytochrome B) rhythms even though both seem to be regulated by similar circadian clock control mechanisms (Hall *et al*. 2002). In addition, cytosolic free calcium (Ca^2+^) and *CAB2::LUC* have different free-running periods under constant red light conditions (Sai & Johnson 1999). Furthermore, *CAT3* (*CATALASE 3*) and *CAB2* also have different periods after entrainment to different temperature cycles, indicating that at least two oscillators with different temperature sensitivities are present within the plant (Michael, Salome & McClung 2003b). It is possible that this uncoupling may confer further temperature-dependent flexibility on the expression patterns of circadian-regulated genes; however, at present, we have no information on whether uncoupling persists under diurnal cycles.

It is possible that multiple oscillators uncouple in response to stress. Due to variation in the geographical locations between *Arabidopsis* accessions and the lack of associated information on collection site environments or collection times, it is difficult to predict the degree of stress different accessions have undergone during natural selection (Hoffmann 2002). While 17 °C is considered to be ‘normal’ for common laboratory strains, it might be stressful for some ‘wild’ *Arabidopsis* lines, therefore, resulting in an early uncoupling of circadian oscillators/loops explaining differentiation between *CCA1* and *LHY* in some accessions (Table 2). Apart from temperature-induced period shortening, the robustness of the *CCA1* and *LHY* rhythmicity was also affected, with *LHY* being affected more severely than *CCA1*. Gould *et al*. (2006) demonstrated that functional *LHY* was more important for *Arabidopsis* at higher temperature, while *CCA1* was more important at lower temperature. Despite this temperature-dependent differentiation, expression of *LHY* was highly down-regulated by the 27 °C treatment in comparison to 17 °C. Expression of CCA1, on the other hand, remained unaffected. Overall, it is intriguing how the importance of LHY transcription increases with its increase in temperature sensitivity (Gould *et al*. 2006).

Our analysis of sequence variation of key clock genes across 14 accessions identified clustering of accessions with specific genotypes (Supporting Information Fig. S4); however, these failed to match with a similar cluster of temperature-dependent clock phenotypes. It is entirely possible that the genetic variation responsible for the phenotypic variation lies outside the limited set of genes investigated. Many recent studies (Gould *et al*. 2006; Portoles & Mas 2010; Salome *et al*. 2010) describe clock components potentially involved in temperature compensation. However, it is more likely to be a feature of the whole network or subset of components rather than a single gene or mechanism (Gould *et al*. 2013). Therefore, it may be difficult in this single-gene analysis approach to identify the molecular sources of variation. Finally, here we have studied variation at the whole gene level. It is possible that at this level single point mutations that are responsible for the phenotype differences have been masked. While this is feasible, what we have found in our analysis is that different accessions tend to have several linked polymorphisms so while a single SNP maybe the causative SNP, it would be associated with a set of linked SNPs. These linked regions would be sufficient to cluster accessions in our trees.

A robust circadian clock with an oscillator period resonant with that of the external L/D cycle enhances growth and survival of *Arabidopsis* plants (Dodd *et al*. 2005). Therefore, initially, we had hypothesized that accessions with a well-buffered clock would perform better when grown under elevated temperatures. Our findings were contrary to this assumption. Accessions with the least temperature-dependent period differences for rhythms of *CCA1* and *LHY* showed the smallest increases in growth with raising temperature. This was not observed for the leaf movement rhythm, even though it was thought to be more temperature compensated. This suggests that the relationship between the temperature, circadian clock and performance is complex. Indeed, several studies have shown the existence of an important link between the circadian clock and plant performance, but this link turns out to be not straightforward. For example, Graf *et al*. (2010) observed that a short period *cca1::lhy* double mutant performed better under 20 h rather than 24 h LD cycle. However, while a short-period *toc1* mutant performs better under 20 h rather than 28 h, its best performance was still at 24 h (Dodd *et al*. 2005; Graf *et al*. 2010). Correlation between weight gain and period is stronger for *LHY::LUC* than for *CCA1::LUC.* Of course, a change in gene transcription does not necessarily directly translate into changes in protein function; however, it is tempting to speculate that transcription of *LHY* has a greater involvement in plant growth regulation at higher temperatures. In addition, temperature influences alternative splicing of *CCA1* and *LHY*, which might be important in growth regulation under changing environmental conditions (James *et al*. 2012). Effects of temperature on transcript and protein stability and function should also be examined.

It would be of great interest to further investigate the effects of the temperature-buffered clock on other aspects of plant performance, for example, chlorophyll content, seed quantity and viability, etc. Flowering time is another aspect of plant physiology that is affected by the circadian clock and temperature, and their relationship across accessions would be valuable to examine. Flowering data was not collected in this study, but it was apparent that flowering time varied between accessions. For the growth performance experiment, all plants were harvested before any accessions bolted; however, it is possible that the growth rate of some plants could have been affected by the vegetative-to-reproductive transition. Nevertheless, our data provides preliminary insights into how the circadian clock could be a major contributor to plant fitness and possibly survival in response to temperature fluctuations in the environment.

An intriguing question is, ‘why is the clock not perfectly buffered against temperature?’, and in fact, ‘why could having a short period at higher temperatures enhance growth?’ One possibility is that the temperature-dependent shortening of period we observe and the altered expression in diurnal cycles may act as an escape mechanism. This would allow the plant to temporally shift temperature-sensitive activities to avoid the warmest and most damaging parts of the day. This idea is further supported by the fact that the desert succulent plant *Kalanchoe fedtschenkoi* also has a clock that oscillates with a short period (Anderson & Wilkins 1989), although it is possible that *K. fedtschenkoi* is not representative of all desert plants. Similarly, the uncoupling mechanism we observe may be a method of allowing temporal shifts for some biological processes while maintaining the coupling of others to a well temperature-buffered 24 h oscillator.

## Supporting Information

**Figure S1.** Normalized average traces for CCA1::LUC (squares) and LHY::LUC (triangles) activity at 17 °C (solid) and 27 °C (empty). Plants were grown on MS agar under 12:12 L/D for 10 daysd before the transfer to 17 °C or 27 °C and continuous light, at which CCA1::LUC and LHY::LUC rhythms were assessed. First 24 h after the transfer to continuous light were not included in period analysis. *n* = 8 for all accessions except Ws-2, where *n* = 2. **Figure S2.** This figure is the sample data as in Figure 5 but non-normalised. Temperature dependent changes in the diurnal expression of clock regulated genes CCA1, LHY, TOC1 and CAB2. Transgenic Col-0 seedlings carrying either CCA1::LUC, LHY::LUC, TOC1::LUC or CAB2::LUC reporter genes were entrained under 12:12 L/D cycles for 7 d, before transferring to 12, 17, or 27 °C and continued 12:12 L/D cycles. The expression pattern for each marker has been graphed separately with plotted lines representing expression at 12 °C (black squares), 17 °C (empty circles) and 27 °C (grey triangles). The plots represent an average of at least 3 independently transformed lines. The experiment was repeated 3 times with the data shown here being a representative of the results gained. **Figure S3.** The phase of maximal expression in 12h L/12h D cycles at 17 and 27 °C was compared to 12 °C for each marker separately. A change in phase occurs at 27 °C for both TOC1 and LHY markers with a ∼2 h phase advance. No phase changes occurred across the temperature range of 12–27 °C for both CAB2 and CCA1. **Figure S4.** Phylogenetic analysis of key circadian clock genes. Gene sequences (1KB upstream plus coding region) were downloaded from the Arabidopsis 1001 genome browser (http://signal.salk.edu/atg1001/index.php) for 14 of the phenotyped accessions. Sequences where aligned using MUSCLE. Plotted in the figure are phylogenetic tree for each of the genes, drawn using the Jukes Cantor model and the Neighbour joining tree-building method. A. unrooted tree for 14 accession and 9 key clock genes. B. Tree for 14 accession and rooted with sequence from Arabidopsis lyrata for CCA1 and PRR7.
