# Supplementary material for: A fast circadian clock at high temperatures is a conserved feature across Arabidopsis accessions and likely to be important for vegetative yield
Source: Plant Cell Environ. 2013 Jul 9;37(2):327–40. doi: 10.1111/pce.12152 (PMC4280891; doi:10.1111/pce.12152)
Supplement: Supplementary file 1 — Figure S1. Normalized average traces for CCA1::LUC (squares) and LHY::LUC (triangles) activity at 17 °C (solid) and 27 °C (empty). Plants were grown on MS agar under 12:12 L/D for 10 daysd before the transfer to 17 °C or 27 °C and continuous light, at which CCA1::LUC and LHY::LUC rhythms were assessed. First 24 h after the transfer to continuous light were not included in period analysis. n = 8 for all accessions except Ws-2, where n = 2. Figure S2. This figure is the sample data as in Figure 5 but non-normalised. Temperature dependent changes in the diurnal expression of clock regulated genes CCA1, LHY, TOC1 and CAB2. Transgenic Col-0 seedlings carrying either CCA1::LUC, LHY::LUC, TOC1::LUC or CAB2::LUC reporter genes were entrained under 12:12 L/D cycles for 7 d, before transferring to 12, 17, or 27 °C and continued 12:12 L/D cycles. The expression pattern for each marker has been graphed separately with plotted lines representing expression at 12 °C (black squares), 17 °C (empty circles) and 27 °C (grey triangles). The plots represent an average of at least 3 independently transformed lines. The experiment was repeated 3 times with the data shown here being a representative of the results gained. Figure S3. The phase of maximal expression in 12h L/12h D cycles at 17 and 27 °C was compared to 12 °C for each marker separately. A change in phase occurs at 27 °C for both TOC1 and LHY markers with a ∼2 h phase advance. No phase changes occurred across the temperature range of 12–27 °C for both CAB2 and CCA1. Figure S4. Phylogenetic analysis of key circadian clock genes. Gene sequences (1KB upstream plus coding region) were downloaded from the Arabidopsis 1001 genome browser (http://signal.salk.edu/atg1001/index.php) for 14 of the phenotyped accessions. Sequences where aligned using MUSCLE. Plotted in the figure are phylogenetic tree for each of the genes, drawn using the Jukes Cantor model and the Neighbour joining tree-building method. A. unrooted tree for 14 accessi [file pce0037-0327-SD1.docx]

**Supplementary figure 1**

Normalized average traces for *CCA1::LUC* (squares) and *LHY::LUC* (triangles) activity at 17°C (solid) and 27°C (empty). Plants were grown on MS agar under 12:12 L/D for 10 days before the transfer to 17°C or 27°C and continuous light, at which *CCA1::LUC* and *LHY::LUC* rhythms were assessed.  First 24 h after the transfer to continuous light were not included in period analysis. N=8 for all accessions except Ws-2, where n=2.


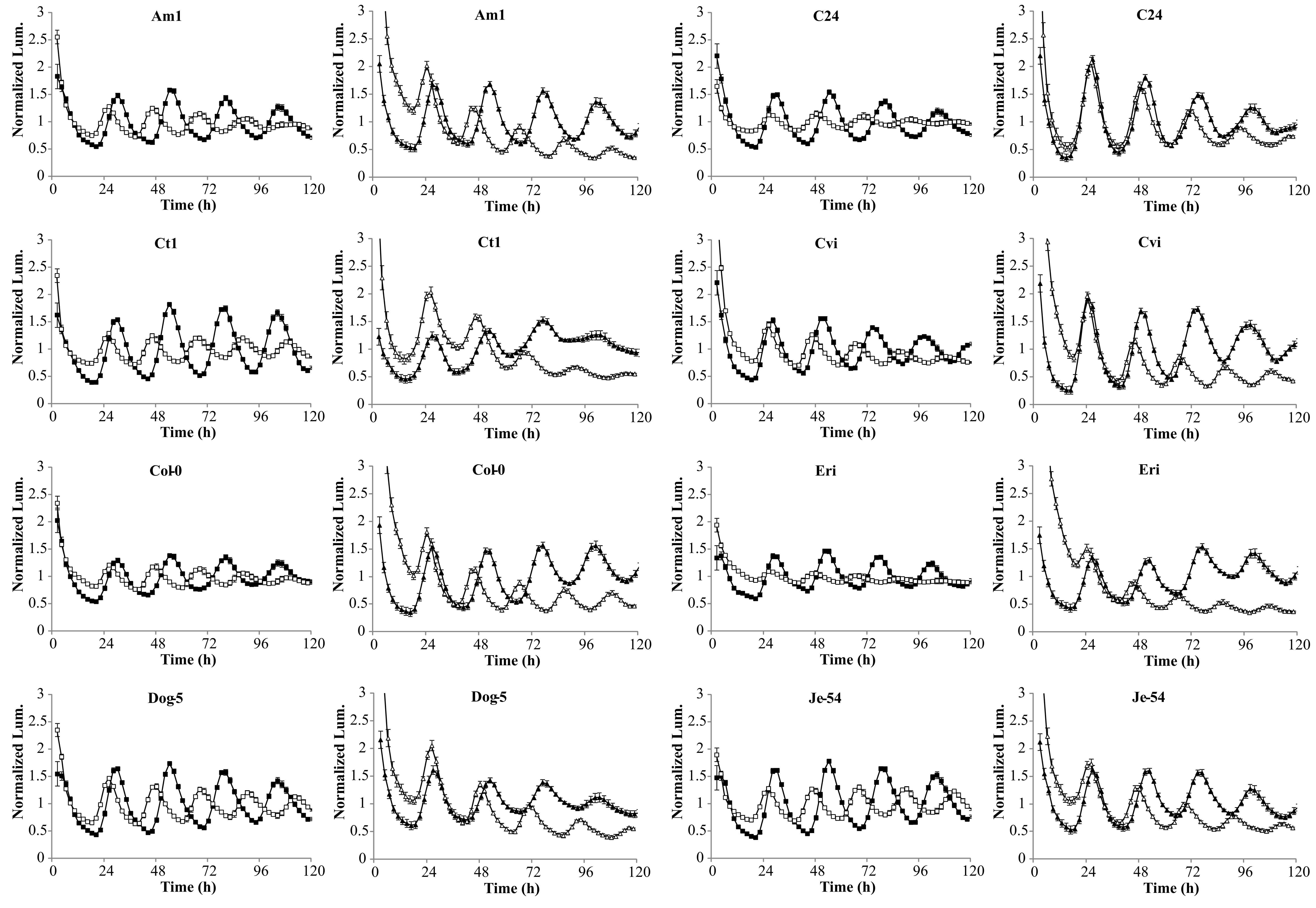


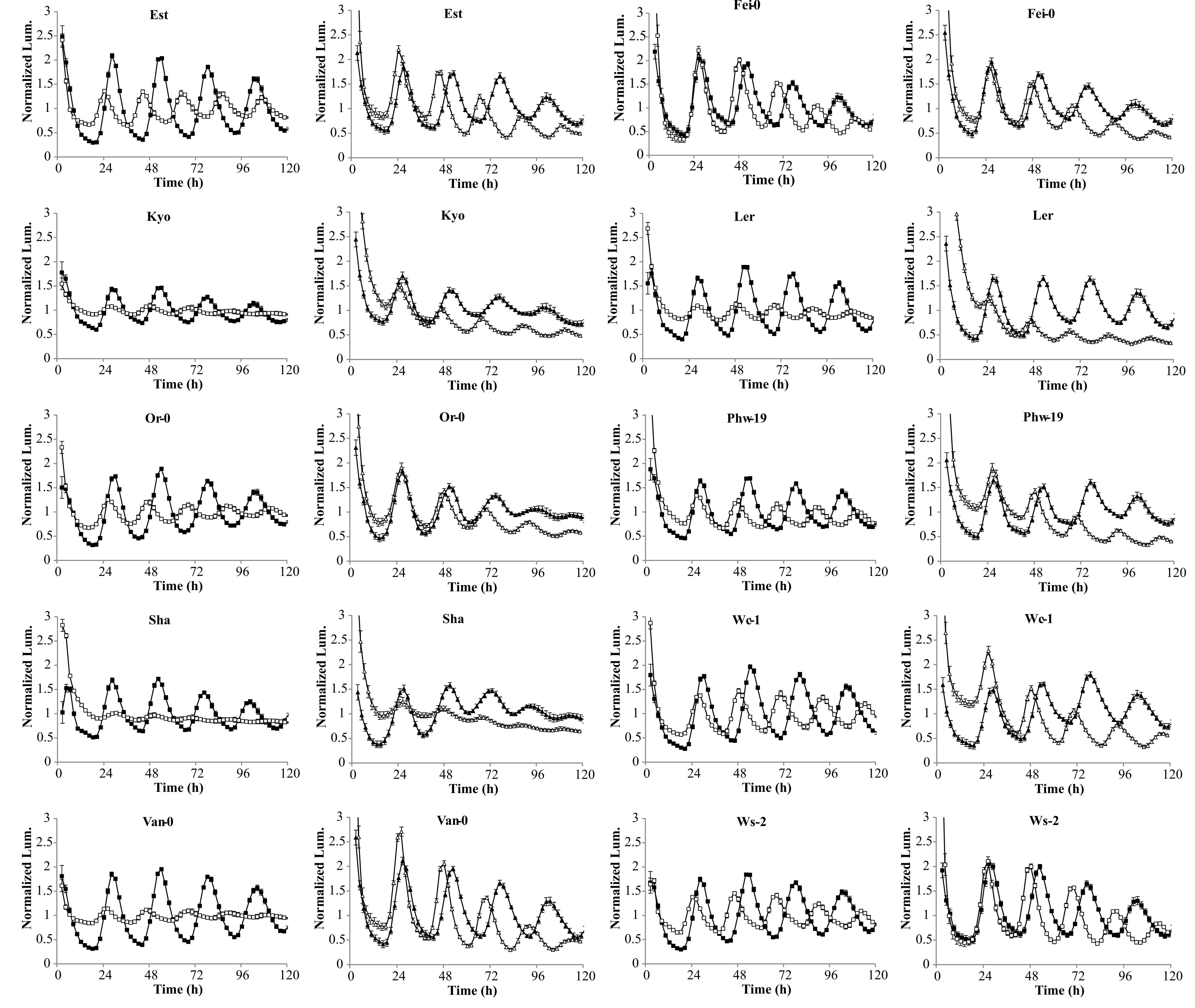


**Supplementary figure 2:**This figure is the sample data as in figure 5 but non-normlised. Temperature dependent changes in the diurnal expression of clock regulated genes *CCA1*, *LHY*, *TOC1* and *CAB2.* Transgenic Col-0 seedlings carrying either *CCA1::LUC, LHY::LUC, TOC1::LUC* or *CAB2::LUC*  reporter genes were entrained under 12:12 L/D cycles for 7 d, before transferring to 12, 17, or 27°C and continued 12:12 L/D cycles. The expression pattern for each marker has been graphed separately with plotted lines representing expression at 12°C (black squares), 17°C (empty circles) and 27°C (grey triangles). The plots represent an average of at least 3 independently transformed lines. The experiment was repeated 3 times with the data shown here being a representative of the results gained.

**Supplementary figure 3**

The phase of maximal expression in 12h L/12h D cycles at 17 and 27°C was compared to 12°C for each marker separately. A change in phase occurs at 27°C for both TOC1 and LHY markers with a ~2hr phase advance. No phase changes occurred across the temperature range of 12-27°C for both CAB2 and CCA1.

**Supplementary figure 4**

Phylogenetic analysis of key circadian clock genes. Gene sequences (1KB upstream plus coding region) were downloaded from the Arabidopsis 1001 genome browser (<http://signal.salk.edu/atg1001/index.php>) for 14 of the phenotyped accessions. Sequences where aligned using MUSCLE. Plotted in the figure are phylogenetic tree for each of the genes, drawn using the Jukes Cantor model and the Neighbour joining tree-building method. A. unrooted tree for 14 accession and 9 key clock genes. B. Tree for 14 accession and rooted with sequence from Arabidopsis lyrata for *CCA1* and *PRR7.*
